# Maternal Health Nurse Competency in Addressing Emergency Obstetric Care: An Assessment in Southern Mozambique

**DOI:** 10.3390/nursrep16080266

**Published:** 2026-07-30

**Authors:** Janeth Dula, Sónia Nhancale, Laurentino Cumbi, Orvalho Augusto, Maria do Rosário Oliveira Martins, Sérgio Chicumbe

**Affiliations:** 1 Health Systems Program, Instituto Nacional de Saúde, Estrada Nacional n°1, Vila de Marracuene, Parcela 3943, Marracuene District, Maputo CEP 0205-02, Maputo Province, Mozambique; sonianhancale77@gmail.com (S.N.); laurentino.cumbi@ins.gov.mz (L.C.);; 2 Global Health and Tropical Medicine (GHTM), Institute of Hygiene and Tropical Medicine, Universidade Nova de Lisboa, 1349-008 Lisbon, Portugal; 3 Institute for Health Metrics and Evaluation, University of Washington, Seattle, WA 98195, USA

**Keywords:** maternal and child health nursing, clinical competence, emergency obstetric care, primary healthcare, Mozambique

## Abstract

**Background:** Quality perinatal health care depends considerably on health workforce competency. Improving Maternal and Child Health Nurse (MCHN) competency is urgently needed in low–middle-income countries, especially to manage key perinatal conditions related to prevalent poor maternal health outcomes, such as pre-eclampsia and hemorrhage. **Objective:** This study assesses the competency of MCHNs in addressing severe pre-eclampsia and antepartum hemorrhage. **Methodology:** A cross-sectional study was conducted from 4 November to 10 December 2022. A structured questionnaire and clinical vignettes concerning severe pre-eclampsia and antepartum hemorrhage were administered to all available MCHNs working at a convenience sample of 80 primary-level health facilities in southern Mozambique. Participant characteristics and competency scores were described using frequency distributions or measures of central tendency, as appropriate. Mixed-effects ordinal regression was used to assess the association between participant characteristics and competency levels. All analyses were performed using Jamovi version 2.6.22 at a 5% significance level. **Results**: Out of 191 participants, most (61.8%, 118) were mid-level MCHNs, with an average age of 33 years (95% CI: 32.05; 34.2). The majority (82.7%, 158) provided prenatal consultation on the day of the interview, and about half (50.7%, 97) knew the national standards for prenatal consultation. MCHNs scoring above 50 percentage points on competency comprised 44.5% (95% CI: 37.4; 51.6%). A higher level of knowledge about guidelines and equipment was associated with increased odds ratios for having a higher-level competency score (OR = 2.21 for “good” vs. reference “poor” competency; 95% CI: 1.07; 4.57, *p* < 0.033). **Conclusions:** About six in 10 MCHNs in Mozambique have poor competency in addressing severe pre-eclampsia and antepartum hemorrhage. The competency level was only associated with knowledge of standards.

## 1. Background

In many contexts, particularly in low- and middle-income countries (LMICs), the quality of pregnancy healthcare is still suboptimal for guaranteeing positive outcomes for women and newborns [1,2]. LMIC health systems rely on Maternal and Child Health Nurses (MCHNs) as a key workforce in determining quality pregnancy healthcare and outcomes [3,4]. Many LMICs face complex health workforce challenges due to insufficient investment, inadequate planning and management, international migration of health workers, and poor adoption and implementation of manpower development policies [5]. Furthermore, MCHNs often lack the necessary competency due to insufficiencies in their pre-service and in-service training, supportive monitoring, and supervision [6].

The Miller competency model is key in assessing the competency of health professionals, and its conceptualization ranges from knowledge (“knows”) to performance (“does”) [7,8,9]. Therefore, assessing competency via clinical simulation is a widely used strategy, as it can standardize performance assessment without changing the safety standards for patients. However, simulation only represents reality and has limitations related to the high costs of setting up standardized scenarios, the need for specialized training to administer, variability in validity, and alignment with real practice. These simulations also depend on the accurate contextualization of the scenarios based on varying clinical cases [10,11,12]. Competency assessment is also performed through the direct observation of workers in real workplace scenarios, and this can provide theoretically high ecological validity; however, it has significant limitations due to inter-observer variability; the high imposed workload for observers; its time-sensitive and -consuming nature; a tendency toward observational bias; high-expenditure roll outs due to organizational factors and logistics [13]; and the extreme difficulty of incorporating relatively infrequent clinical events. Clinical vignettes are an additional well-established approach to assessing competency; they are standardized and cost-effective, allowing for exploration of variations in practice and cognitive heuristics. However, clinical vignettes have limitations in their external validity when measuring real behaviors; this depends on using simple constructs while also maintaining methodological rigor. Nonetheless, vignettes are well suited and widely used for competency assessment [14,15,16].

Health workforce development has been prioritized in LMICs to strengthen their health systems, with an emphasis on primary care, and as a means to implement innovations and technologies to improve health. These efforts have often focused on rural areas [7]. Actions derived from health workforce development plans in LMICs are also crucial for reducing health inequalities and improving health outcomes for mothers and children, especially by promoting increased availability of competent obstetric practitioners, specifically MCHNs.

As of December 2019, Mozambique had 58,124 healthcare workers, 55% of whom were directly involved in frontline health service delivery and 45% in support and administrative roles. Mozambique has 18 public pre-service nurse training institutions and over 100 private health sciences institutes, with an average of 3000 graduates per year [8,9]. Anecdotally, the quality of training, particularly in the private sector, has become a concern due to a lack of standardization and proper accreditation [10]. A 2023 study examining MCHN availability and distribution in Mozambique identified an unequal distribution of healthcare professionals, with urban areas benefiting more than rural areas [9].

The clinical competency of healthcare professionals in Mozambique is perceived as limited and significantly variable, with a growing need to improve training and clinical skills. Mozambique’s policies emphasize increasing the availability and equity of competent professionals, retaining professionals in their occupational areas, and enhancing satisfaction and competency levels for the provision of humanized, quality services [8]. A notable gap remains in the literature regarding the clinical competency of MCHNs in managing critical obstetric emergencies. Existing studies are geographically limited [17,18,19] and predominantly focus on knowledge-based assessments [20,21,22]. This limitation is critical for high-burden conditions such as pre-eclampsia and obstetric hemorrhage, which continue to drive maternal mortality.

In African countries, there is a general evidence gap regarding the levels of—and factors associated with—health workforce competency in providing healthcare, with Mozambique being no exception. The present study addresses this evidence gap by providing a context-specific, multidimensional assessment of MCHN competency in southern Mozambique, thereby offering novel, practice-oriented evidence to inform workforce strengthening, competency-based training, and quality improvement strategies. Understanding MCHN competency levels and factors is essential to informing continuous health improvement and to the quality of healthcare provided to pregnant women and newborns. Specifically, this study aimed to assess MCHN competency and associated factors in providing primary-level emergency obstetric care in Mozambique.

## 2. Methodology

### 2.1. Study Design

This is an observational, quantitative, and cross-sectional analytical study. Data collection occurred between 4 November and 10 December 2022.

### 2.2. Setting

The study was conducted in all four southern Mozambique provinces, at 80 public health facilities (HFs), distributed as follows: Inhambane (10 HF), Gaza (31 HF), Maputo (24 HF), and Maputo City (14 HF). Fourteen health facilities were in urban areas.

### 2.3. Population

The target population was MCHNs working at primary health centers in southern Mozambique with at least six months of working experience. The six-month cutoff is the minimum clinical experience time needed for a participant to be exposed to HF patterns and factors related to the provision of perinatal healthcare, such as medicine and equipment availability.

### 2.4. Sample Size and Sampling Methods

This cross-sectional study was embedded in a primary study, a randomized controlled trial (RCT) conducted in southern Mozambique testing whether a compound intervention on HF management could increase the number and quality of ANC (the study’s results have yet to be published). The RCT treatment and control groups each consisted of 40 HFs; this allowed for the detection of a minimum 10% increase in the number of ANC visits, with 80% power and a significance level of 5%. The sampling was drawn from a list of HFs previously ranked using the average number of monthly ANC visits entered in the master facility list of the Ministry of Health. This study included all 80 HFs under study in the RCT. At each HF, a census of all MCHNs working in the facility was performed. The mode of MCHNs per HF in Mozambique was two; thus, we expected at least 160 MCHNs to be included in this study. According to the 2018 HF inventory (SARA 2018) [11], the provinces of southern Mozambique had 391 public primary-care HF capable of providing antenatal consultation (ANC). Therefore, this study used a convenience sample of 20.5% of the total HFs that perform ANC in southern Mozambique and a census of MCHNs in all 80 HFs.

### 2.5. Variables

#### 2.5.1. Dependent Variable

The dependent variable is a competency score (scale: 0–100 percentage points) assessed using two clinical vignettes pertaining to severe pre-eclampsia and antepartum hemorrhage. Individual scores were consecutively transformed and categorized into four levels (D—poor/insufficient < 50%, C—sufficient < 65%, B—good < 90%, A—excellent ≥ 90%), per classifications routinely used at health professional training institutes in Mozambique.

#### 2.5.2. Explanatory Variables

The explanatory variables are province; area of residence (rural vs. urban); professional category (basic, mid, higher level of education); age; self-reported fit for ANC; last date of ANC provision; years of clinical experience; and a compound index of knowledge of ANC standards and equipment, which was also categorized in four levels (D—poor/insufficient < 50%, C—sufficient < 65%, B—good < 90%, A—excellent ≥ 90%), per classifications routinely used at health professional training institutes in Mozambique.

### 2.6. Data Collection Tool

The interviews were conducted by trained surveyors using structured but indirect questions about the explanatory variables. Clinical vignettes were used to measure competency scores in managing two critical maternal emergencies: severe pre-eclampsia and antepartum hemorrhage. With the participation of Ministry of Health staff, the study’s data collection tools were adapted from similar studies carried out in Africa [12].

### 2.7. Vignettes and Scores

This study’s clinical vignettes describe two cases of obstetric emergency: Case A describes a pregnant woman with signs and symptoms of severe pre-eclampsia, and Case B presents a case of severe antepartum hemorrhage. Each vignette is divided into sections to elicit MCHN approaches to diagnosis and management. Respondents provided open responses and were asked if they would do anything else until they explicitly said “no”; the trained interviewer marked a checklist accordingly.

The vignette response options included best practices, reasoning and actions related to diagnosis, and initial management of obstetric emergencies, aligned with the WHO Pregnancy, Childbirth, Postpartum, and Newborn Healthcare guidelines for primary care. The checklist also included additional management actions, such as supplemental oxygen administration, that can be performed in a well-functioning first-line or referral HF (Supplementary Material—Table S1).

Scores were computed by assigning one point to each correct option for the specific clinical case. For interventions that would be performed only or mainly at referral facilities, rather than at a primary level of care (health centers)—specifically, ultrasound, blood transfusion, supplemental oxygen and caesarean delivery—respondents received one point if the action was not available at their facility and if the respondent said they would refer the woman to a facility capable of intervening with, for example, a caesarean delivery (Supplementary Material—Table S1). Considering vignettes A and B, the scores for the diagnostic approaches and management section were 10 points each, to a maximum of 20 points. The overall competency score was computed as the mean of the Case A and B scores. The overall competency score was then categorized per national health sector educational norms: D—insufficient/poor/fail (<50%), C—sufficient (<65%), B—good (<90%), and A—excellent (≥90%).

### 2.8. Data Analysis

Participant demographics, training, and knowledge about prenatal care standards are described using absolute and relative frequencies. The frequency distribution of MCHNs per competency level is described using absolute and relative frequencies. Ordinal regression was used to analyze the association between an ordinal dependent variable and one or more explanatory variables [13]. Since the competency score was ranked from lowest (D) to highest (A), we employed simple (bivariate) mixed-effects ordinal regression analysis to assess factors associated with achieving the highest competency level. The random effects were set at the HF level considering the MCHN cluster. If explanatory variables in the bivariate analysis were associated with the highest competency scores at *p* ≤ 0.20, we included those in the mixed-effects multiple ordinal regression analysis at a significance level of 5%. Unadjusted and adjusted odds ratios (AORs) and their 95% confidence intervals (CIs) are reported for the associations. All analyses were performed using Jamovi 2.6.22.

### 2.9. Ethical Considerations

The participants informedly and freely consented to participate in this study. This study followed national and international ethical standards, especially the Declaration of Helsinki, and was approved by Mozambique’s National Bioethics Committee (reference 761/CNBS/2021).

## 3. Results

Participants (N = 191) were all female, with a mean age of 33.1 (95% CI: 32.05–34.19) years, and 57% (n = 109) were from HFs in rural areas. The majority (61%; n = 118) had mid-level (three years college) training, and 29% were high-level (five years university) trained nurses. Only 13% (n = 25) of participants had less than one year of working experience, and 32% (n = 61) had worked for more than 6 years. The participant profiles are described in Table 1.

More than two-thirds (82%; n = 157) self-assessed and reported being skilled at providing ANC, and 84% (n = 161) stated that they indeed provided ANC. Additionally, 83% (n = 158) had performed ANC on the day of the interview.

**Knowledge and Competency**

Overall, about half (51%; n = 97) of the MCHNs had knowledge of the essential standards and equipment required to provide ANC. In Case A—Severe Pre-eclampsia, 59.7% (95% CI: 52.7; 66.7%) of MCHNs scored D—insufficient. In Case B—Antepartum Hemorrhage, 59.2% (95% CI: 52.1; 66.2%) of MCHNs scored D—insufficient. In overall competency, 55.5% (95% CI: 48.4; 62.6%) of MCHNs scored D—insufficient, 44.5% (95% CI: 37.4; 51.6%) scored above D (C, B, or A), and 9.4% (95% CI: 5.2; 13.6%) scored A—excellent.

Knowledge of guidelines and equipment was the only factor marginally associated with increased odds of competency, at a significance level of 5%. Compared with a score of D, a score of B was associated with significantly higher odds of achieving high competency (OR = 2.2; 95% CI: 1.07–4.57; *p* = 0.033) (Table 2).

## 4. Discussion

To the best of our knowledge, this is the first study assessing MCHN competency in providing emergency obstetric care in Mozambique. The provision of high-quality care, delivered by well-trained professionals, is essential to ensuring favorable pregnancy outcomes and a positive perinatal experience [23]. Healthcare providers’ clinical competency plays a crucial role in the early identification of obstetric risks, the timely implementation of best practices and interventions, and the promotion of maternal and neonatal health.

A substantial proportion (55.5%) of MCHNs scored poorly in competency for the selected Emergency Obstetric Care (EmOC) scenarios, corroborating the results of similar studies conducted in LMICs, such as Ghana (55%) [12] and Bangladesh (50%) [14], where about half of MCHNs had low levels of EmOC competency. MCHN knowledge of ANC standards and equipment was associated with an increased odds ratio of scoring a higher level of competency. Evidence demonstrates that a nurse’s knowledge of clinical standards and medical equipment determines the quality of care [12,15,16,24]. As demonstrated in Ethiopia [15], healthcare professionals with greater expertise exhibit superior neonatal care practices, and these findings suggest that competency is essential to ensuring high-quality perinatal outcomes [15].

Theoretically, a positive association can be expected between several factors—such as academic level, length of service experience and in-service training, and MCHN competency score—but this hypothesis was not confirmed in this study. Other scholars suggest that the observed dissociation between potential logical factors and competency is due to the prevailing poor quality of pre-service training at all levels, as well as the low impact of continuous training [25]. A cohort study conducted in India [26] demonstrated that trained obstetric nurses, when subjected to regular monitoring, audits, and integration into reliable referral facilities, can provide high-quality care. A study in Kenya [5] found that better-equipped healthcare units and more extensively trained nurses positively impact nursing performance in maternal and neonatal care. Other studies [18,27,28,29] have highlighted the need to invest in short, competency-based training programs for EmOC to foster continuous improvement in healthcare professionals’ knowledge and skills while also driving meaningful changes in clinical practice.

Of the MCHNs interviewed in our study, about 83% provided ANC on the day of the interview, and 82% felt they were competent in performing obstetric care. This result suggests that the perceptions of professionals about their own capabilities do not necessarily align with demonstrated competency. Only 51% were familiar with the national standards for prenatal care, which corroborates a study carried out in Tanzania [17] with similar findings. Our findings and interpretations are consistent with the results of studies conducted in Turkey [30] and Ghana [18], which demonstrated that maternal healthcare nurses consider themselves competent and proficient but exhibit significant gaps in managing relevant obstetric conditions.

Women and mid-level MCHNs comprise the majority of frontline obstetric care providers, with an average age of 33 (SD = 7.5). The average age of MCHNs in southern Mozambique is considerably higher when accounting for the predominance of youth in the demographic structure of the country’s population [19], and three years is the duration of pre-service training for this cadre. In addition, a third of the interviewed MCHNs had more than 6 years of experience as obstetric healthcare providers. Altogether, these indicators raise important questions regarding the pattern of recruitment for training nurses, as well as its impact on service delivery. We hypothesize that candidates for pre-service training as MCHNs are unlikely to enroll at a young age, as suggested by the variation between the relatively small proportion of MCHNs with more than 6 years of experience and the relatively advanced mean age (24% variation coefficient). A distinct pattern was observed in Tanzania [17] and Ethiopia [21], where the majority of MCHNs had more than 5 years of professional experience—63% and 42%, respectively. Results from other sub-Saharan African countries [16] corroborate our findings, showing that most nurses involved in maternal and newborn health have less than 10 years’ work experience.

The finding that MCHNs in Mozambique are all women, are relatively advanced in age, have received predominantly mid-level training, and have only brief clinical experience is context-specific but policy-relevant. Our contextual knowledge shows that the geographical locations of public pre-service MCHN training institutions are predominantly urban areas, far from the rural backgrounds of most students (aspirant MCHNs). This may present an unobserved, yet likely, obstacle to achieving adequate competency in pre-service training. The need to travel to and/or reside in urban educational establishments imposes social, economic, and logistical challenges, particularly for women from rural areas and with socioeconomically disadvantaged backgrounds [31]. Although not directly assessed in the study, these contextual factors may be the underlying reason for women’s late entry into professional training, impacting not only the demographics of frontline MCHNs in this country but also their attainable competency levels.

### 4.1. Study Limitations

This study is cross-sectional and was carried out at a limited number of primary HF and in the context of Mozambique, resulting in a relatively small sample of MCHNs. However, our HF sample is large in programmatic terms, as it includes almost all nurses available at the selected HFs and a substantial number (20%) of existing primary health facilities in southern Mozambique.

Our measurement of competency levels using a limited number of clinical vignettes could have been strengthened by direct observation of MCHN practices; however, complications such as antenatal hemorrhage and pre-eclampsia are relatively infrequent in daily practice, making data collection through direct observation challenging if not unfeasible. The vignettes could have been adapted to Mozambique by conducting a pilot study to ensure the cultural adaptability of their translations and to match the levels of technical language within the Mozambican context. Nonetheless, this is a well-standardized method for assessing competency in health workforces. In addition, the tools were assessed by the Ministry of Health for alignment with national norms and training materials on antenatal hemorrhage and pre-eclampsia, which are based on WHO standards. Another strength of this study is that the clinical vignettes focused on severe pre-eclampsia and antepartum hemorrhage, as these are the most relevant perinatal complications in this context, requiring prompt decision-making and management by competent nurses in primary HFs.

Although we believe that our exploration of factors associated with competency levels has revealed potential areas for policy improvement, the small sample size of MCHNs and the clustering of competency scores at the HF level may limit the inferential analysis. To minimize this concern, we employed bivariate mixed-effects ordinal regression; however, the outputs had low variance, as explained by HF potential clustering.

### 4.2. Implications

Improving MCHN competency in Mozambique is an urgent need and may require implementing targeted, continuous support and supervision programs, including mentorship programs and the development of professional support networks. Equally essential is rolling out practices that can strengthen pre-service and in-service obstetric skills education. Thus, we recommend revising and enhancing existing training initiatives, including the content and training modalities, to ensure MCHNs have access to adequate, up-to-date training materials and competency, eliciting pre-service and continuous education. Furthermore, this study highlights the importance of implementing continuous competency monitoring systems in Mozambique’s health sector.

## 5. Conclusions

While most MCHNs self-reported having competency for ANC, objective assessments revealed significant gaps in addressing EmOC. About six in ten MCHNs demonstrated gaps in the ability to address severe antepartum hemorrhage and pre-eclampsia, which are major determinants of poor perinatal outcomes. Knowledge of guidelines and equipment used to provide ANC was associated with higher EmOC competency levels. The results underscore the urgent need for programs targeting MCHN competency gaps, including, but not limited to, effective training modalities for frontline healthcare providers.

## Figures and Tables

**Table 1:** Profile of Maternal and Child Health Nurses in public health centers, southern Mozambique (n = 191).

Descriptives					
Characteristics	N	Missing	Estimate	95% Confidence Interval	
				Lower	Upper
**Sex (n, %)**					
Female	191	0	100	100	100
**Age in years (mean)**	191	0	33.12	32.05	34.19
**Maternal and Child Health Nurses Category (n, %)**					
Basic	191	0	3.66	0.98	6.35
Mid/Intermediate	191	0	61.78	54.83	68.73
Higher	191	0	28.27	21.83	34.72
**Province (n, %)**					
Maputo	191	0	30.37	23.79	36.95
Gaza	191	0	37.17	30.26	44.09
Inhambane	191	0	12.57	7.82	17.31
**Area**					
Rural	191	0	57.07	49.99	64.15
**Years of work experience (n, %)**					
1 Year	191	0	13.09	8.26	17.92
1–3 Years	191	0	29.84	23.30	36.39
4–6 Years	191	0	25.13	18.92	31.34
more 6 Years	191	0	31.94	25.27	38.61
**Service providing antenatal care (n, %)**					
Yes	191	0	84.29	79.09	89.50
**Provided prenatal care on the day of the interview (n, %)**					
Yes	191	0	82.72	77.31	88.13
**Self-reported ability to provide antenatal care (n, %)**					
Yes	191	0	82.20	76.73	87.67
**Knowledge of standards and essential equipment for a prenatal consultation (n, %)**					
Yes	191	0	50.73	47.58	53.88
**Competency for pre-eclampsia cases (n, %)**					
Insufficient	191	0	59.69	52.67	66.71
Sufficient	191	0	17.80	12.33	23.28
Good	191	0	14.14	9.15	19.12
Excellent	191	0	8.38	4.41	12.34
**Competency for cases of antepartum hemorrhage (n, %)**					
Insufficient	191	0	59.16	52.13	66.20
Sufficient	191	0	18.32	12.79	23.86
Good	191	0	14.66	9.60	19.72
Excellent	191	0	7.85	4.00	11.70
**Competency of nurses to provide emergency obstetric care (n, %)**					
Insufficient	191	0	55.50	48.39	62.61
Sufficient	191	0	20.42	14.65	26.19
Good	191	0	14.66	9.60	19.72
Excellent	191	0	9.42	5.24	13.61
Sufficient plus (CBA)	191	0	44.50	37.39	51.61

**Table 2:** Factors associated with competency in providing emergency obstetric care in southern Mozambique (n = 191).

					Exp(B) 95% Confidence Intervals			
Names	Effect	Estimate	SE	Exp(B)	Lower	Upper	z	*p*
(Threshold)	D|C	0.243	0.1554	1.275	0.94	1.73	1.563	0.118
(Threshold)	C|B	1.2108	0.1967	3.356	2.282	4.94	6.154	<0.001
(Threshold)	B|A	2.3553	0.2848	10.542	6.033	18.42	8.271	<0.001
**Age**	Age	−0.0185	0.0243	0.982	0.936	1.03	−0.762	0.446
(Threshold)	D|C	0.237	0.149	1.268	0.947	1.7	1.593	0.111
(Threshold)	C|B	1.167	0.173	3.212	2.288	4.51	6.745	<0.001
(Threshold)	B|A	2.285	0.251	9.821	6.004	16.06	9.098	<0.001
0 = Another (basic and higher)	Ref							
**Maternal and Child Health Nurses Category = Intermediate**	0–1	−0.158	0.285	0.854	0.488	1.49	−0.553	0.58
(Threshold)	D|C	0.102	0.312	1.11	0.601	2.04	0.328	0.743
(Threshold)	C|B	1.03	0.322	2.8	1.491	5.26	3.203	0.001
(Threshold)	B|A	2.143	0.373	8.52	4.101	17.71	5.742	<0.001
0 = Another (intermediate and higher)	Ref							
**Maternal and Child Health Nurses Category = Basic**	1–0	0.265	0.617	1.3	0.389	4.37	0.429	0.668
(Threshold)	D|C	0.183	0.157	1.201	0.883	1.63	1.167	0.243
(Threshold)	C|B	1.115	0.185	3.051	2.123	4.39	6.025	<0.001
(Threshold)	B|A	2.232	0.265	9.318	5.544	15.66	8.425	<0.001
0 = Another (Gaza and Inhambane)	Ref							
**Maputo Province**	0–1	−0.218	0.305	0.804	0.442	1.46	−0.714	0.475
(Threshold)	D|C	0.211	0.164	1.24	0.895	1.7	1.287	0.198
(Threshold)	C|B	1.147	0.195	3.15	2.149	4.61	5.887	<0.001
(Threshold)	B|A	2.272	0.273	9.7	5.683	16.54	8.335	<0.001
0 = Another (Maputo and Inhambane)	Ref							
**Gaza Province**	1–0	0.121	0.323	1.13	0.6	2.12	0.375	0.708
(Threshold)	D|C	0.243	0.148	1.28	0.955	1.7	1.65	0.1
(Threshold)	C|B	1.176	0.172	3.24	2.313	4.54	6.84	<0.001
(Threshold)	B|A	2.294	0.25	9.91	6.072	16.18	9.17	<0.001
0 = Urban	Ref							
**Rural**	1–0	0.3	0.283	1.35	0.776	2.35	1.06	0.288
(Threshold)	D|C	0.275	0.214	1.32	0.866	2	1.286	0.198
(Threshold)	C|B	1.231	0.239	3.43	2.145	5.47	5.155	<0.001
(Threshold)	B|A	2.366	0.31	10.66	5.808	19.56	7.64	<0.001
0 = Another	Ref							
**Years of work experience = 1 year**	0–1	0.117	0.43	1.12	0.484	2.61	0.273	0.785
(Threshold)	D|C	0.2322	0.158	1.261	0.926	1.72	1.471	0.141
(Threshold)	C|B	1.1596	0.18	3.189	2.24	4.54	6.435	<0.001
(Threshold)	B|A	2.2747	0.256	9.725	5.893	16.05	8.901	<0.001
0 = Another	Ref							
**Years of work experience = 1–3 years**	1–0	−0.0572	0.303	0.944	0.521	1.71	−0.189	0.85
(Threshold)	D|C	0.2363	0.164	1.267	0.919	1.75	1.445	0.148
(Threshold)	C|B	1.1634	0.185	3.201	2.229	4.6	6.305	<0.001
(Threshold)	B|A	2.2788	0.259	9.765	5.875	16.23	8.791	<0.001
0 = Another	Ref							
**Years of work experience = 4–6 years**	1–0	−0.0661	0.315	0.936	0.505	1.73	−0.21	0.834
(Threshold)	D|C	0.146	0.192	1.16	0.795	1.69	0.762	0.446
(Threshold)	C|B	1.075	0.208	2.93	1.947	4.41	5.156	<0.001
(Threshold)	B|A	2.191	0.275	8.94	5.213	15.33	7.958	<0.001
0 = No	Ref							
**Service providing antenatal care**	0–1	0.22	0.372	1.25	0.601	2.58	0.592	0.554
(Threshold)	D|C	0.122	0.185	1.13	0.786	1.62	0.66	0.509
(Threshold)	C|B	1.052	0.202	2.864	1.927	4.25	5.207	<0.001
(Threshold)	B|A	2.168	0.27	8.744	5.15	14.85	8.028	<0.001
0 = No	Ref							
**Provided prenatal care on the day of the interview**	1–0	−0.305	0.358	0.737	0.366	1.49	−0.854	0.393
(Threshold)	D|C	0.149	0.185	1.16	0.807	1.67	0.803	0.422
(Threshold)	C|B	1.132	0.213	3.1	2.042	4.71	5.307	<0.001
(Threshold)	B|A	2.287	0.294	9.85	5.536	17.52	7.784	<0.001
Knowledge of standards and essential equipment for a prenatal consultation—insufficient	Ref							
**Knowledge of standards and essential equipment for a prenatal consultation—sufficient**	C–D	0.614	0.392	1.85	0.857	3.98	1.567	0.117
**Knowledge of standards and essential equipment for a prenatal consultation—good**	B–D	0.792	0.371	2.21	1.066	4.57	2.133	0.033
**Knowledge of standards and essential equipment for antenatal consultation—excellent**	A–D	0.131	0.573	1.14	0.371	3.5	0.228	0.82

## Data Availability

The datasets used and/or analyzed during the current study are available from the corresponding author upon reasonable request.

## Supplementary Materials

The following supporting information can be downloaded at https://www.mdpi.com/article/10.3390/nursrep16080266/s1, Table S1: Vignette cases and construction of vignette score.

## Public Involvement Statement

There was no public involvement in any aspect of this research.

## Guidelines and Standards Statement

This manuscript was drafted against the STROBE (Strengthening the Reporting of Observational Studies in Epidemiology) Statement [32] for observational research. A complete list of reporting guidelines can be accessed via the EQUATOR Network: https://www.equator-network.org/ (accessed on 13 July 2026).

## Abbreviations

The following abbreviations are used in this manuscript:

AOR	Adjusted odds ratio
ANC	Prenatal care
EmOC	Emergency obstetric care
HF	Health facilities
MISAU	Ministry of Health of Mozambique
MCHN	Maternal and Child Nurse
LMICs	Low- and middle-income countries
95% CI	95% confidence intervals
WHO	World Health Organization
OR	Odds ratio
SIS-MA	Data from the Mozambique Health Information System
