# Supplementary material for: Maternal Health Nurse Competency in Addressing Emergency Obstetric Care: An Assessment in Southern Mozambique
Source: Nurs Rep. 2026 Jul 30;16(8):266. doi: 10.3390/nursrep16080266 (PMC13516758; doi:10.3390/nursrep16080266)
Supplement: Supplementary file 1 [file nursrep-16-00266-s001.zip › nursrep-4325672-supplementary.pdf]

Supplementary Materials 1

Table S1. Vignette cases and construction of vignette score.

Vignette action	Vignette score
**Case A.** Section 1. A 26-year-old woman who is 7 months pregnant comes in complaining of headaches, blurred vision, and epigastric pain, and her face looks swollen. In this facility, what would you usually do to establish a diagnosis? Measure the woman's blood pressure*	
Check her urine for protein*	+1 point if mentioned
Check her reflexes*	+1 point if mentioned
Check foetal heart rate*	+1 point if mentioned
Refer to other health facility* or	+1 point if at least one out of two mentioned
Call specialist or respondent is a specialist (i.e., a doctor)	
	Section maximum: 5 points
**Case A.** Section 2. On examination, she had a blood pressure of 170/120 mm Hg, 3+ protein in her urine and brisk reflexes. Measure the woman's blood pressure*	
Giving antihypertensive drug, e.g., hydralazine, labetalol or nifedipine*	+1 point if mentioned
Give magnesium sulphate or, if not available, diazepam*	+1 point if mentioned
Have somebody stay with her all the time in case she starts having seizures*	+1 point if mentioned
Plan for delivery within the next 24 hours, or	+1 point if at least one out of two mentioned
Refer to other health facility*	
	Section maximum: 4 points
**Case B.** Section 1. A 35-year-old woman who is 8 months pregnant comes to this facility because she has started to bleed heavily vaginally. She has no contractions and does not complain of any pain. In this facility, what would you usually do to establish a diagnosis?	
Check the woman's vital signs*	+1 point if mentioned
Check foetal heart rate	+1 point if mentioned
Perform abdominal examination	+1 point if mentioned
Do not perform vaginal examination*	+1 point if mentioned
Refer to a facility where caesarean delivery can be performed*, or	+1 point if at least one out of two mentioned
Call specialist or respondent is a specialist (i.e., a doctor)	

	Section maximum: 5 points
**Case B.** Section 2. The woman has a feeble pulse at 120/min, her systolic blood pressure is 85, and she is pale, sweating, and breathing rapidly at 30 breaths per minute. Foetal heart sound is normal. There is no pain on abdominal examination. She is still bleeding bright red blood vaginally. You suspect placenta praevia and, therefore, do not perform a vaginal examination.<br><br>How would such a patient be managed now?	
Elevate legs to increase return of blood to the heart*	+1 point if mentioned
Give intravenous fluids rapidly*	+1 point if mentioned
Give oxygen by mask or nasal cannula, or	+1 point if at least one out of two mentioned
If oxygen unavailable, refer to a facility where caesarean delivery can be performed	
Perform ultrasound to confirm diagnosis, or	+1 point if at least one out of two mentioned
If ultrasound unavailable, refer to a facility where caesarean delivery can be performed	
Give blood transfusion	+1 point if at least one out of two mentioned
If blood transfusion unavailable, refer to a facility where caesarean delivery can be performed	
Prepare for caesarean delivery, or refer to a facility where caesarean delivery can be performed*	+1 point if at least one out of two mentioned
	Section maximum: 6 points
**Cases A and Case B**	Total maximum: 20 points
*Vignette actions mentioned in WHO Pregnancy, Childbirth, Postpartum and Newborn care.21	

Model adapted from the article by Lohela et al [18].
